# Global DNA methylation profiling uncovers distinct methylation patterns of protocadherin alpha4 in metastatic and non-metastatic rhabdomyosarcoma

**DOI:** 10.1186/s12885-016-2936-3

**Published:** 2016-11-14

**Authors:** L. Tombolan, E. Poli, P. Martini, A. Zin, C. Millino, B. Pacchioni, B. Celegato, G. Bisogno, C. Romualdi, A. Rosolen, G. Lanfranchi

**Affiliations:** 1 Department of Biology, University of Padova, Padova, Italy; 2 C.R.I.B.I.-Biotechnology Centre, University of Padova, Padova, Italy; 3 Institute of Pediatric Research, IRP, Padova, Italy; 4 Department of Women’s and Children’s Health, Hematology Oncology Division, University of Padova, Padova, Italy; 5 Department of Women’s and Children’s Health, University of Padova, Padova, Italy

**Keywords:** Rhabdomyosarcoma, *PCDHA4*, Microarray, DNA methylation, Epigenetics

## Abstract

**Background:**

Rhabdomyosarcoma (RMS), which can be classified as embryonal RMS (ERMS) and alveolar RMS (ARMS), represents the most frequent soft tissue sarcoma in the pediatric population; the latter shows greater aggressiveness and metastatic potential with respect to the former. Epigenetic alterations in cancer include DNA methylation changes and histone modifications that influence overall gene expression patterns. Different tumor subtypes are characterized by distinct methylation signatures that could facilitate early disease detection and greater prognostic accuracy.

**Methods:**

A genome-wide approach was used to examine methylation patterns associated with different prognoses, and DNA methylome analysis was carried out using the Agilent Human DNA Methylation platform. The results were validated using bisulfite sequencing and 5-aza-2′deoxycytidine treatment in RMS cell lines. Some in vitro functional studies were also performed to explore the involvement of a target gene in RMS tumor cells.

**Results:**

In accordance with the Intergroup Rhabdomyosarcoma Study (IRS) grouping, study results showed that distinct methylation patterns distinguish RMS subgroups and that a cluster of protocadherin genes are hypermethylated in metastatic RMS. Among these, *PCDHA4,* whose expression was decreased by DNA methylation, emerged as a down-regulated gene in the metastatic samples. As *PCDHA4*-silenced cells have a significantly higher cell proliferation rate paralleled by higher cell invasiveness, *PCDHA4* seems to behave as a tumor suppressor in metastatic RMS.

**Conclusion:**

Study results demonstrated that DNA methylation patterns distinguish between metastatic and non-metastatic RMS and suggest that epigenetic regulation of specific genes could represent a novel therapeutic target that could enhance the efficiency of RMS treatments.

**Electronic supplementary material:**

The online version of this article (doi:10.1186/s12885-016-2936-3) contains supplementary material, which is available to authorized users.

## Background

Rhabdomyosarcoma (RMS) represents the most frequent soft tissue sarcoma in pediatric patients. The two main histological subtypes of RMS tumors, alveolar RMS (ARMS) and embryonal RMS (ERMS), have distinct molecular and clinical profiles. The former, in fact, is characterized by more aggressive behavior and a higher tendency to present with signs of metastatic disease at diagnosis and to relapse after treatment [1]. Approximately 80 % of ARMS harbor the reciprocal chromosomal translocation t(2;13) (q35;q14) or the less common variant translocation t(1;13)(p36;q14) in which *PAX3* and *FOXO1*, or *PAX7* and *FOXO1* genes, respectively, are juxtaposed [2]. The latter subtype, instead, is not characterized by specific genetic aberrations except for a loss of heterozygosity at 11p15, which could mean that this region contains tumor suppressor genes.

Over the past decade many genome-wide studies have demonstrated that fusion-positive and negative RMS present different gene expression signatures [3, 4]. Despite the low rate of gene mutations shown by RMS, recent genomic studies have revealed that recurrent mutations in several key genes characterize different RMS subtypes. In particular, mutations in receptor tyrosine kinase/*RAS/PIK3CA* and *FGFR* signaling predominately affect fusion negative tumors [5]. The presence of metastasis at diagnosis represents the strongest predictor of poor outcome, and the 5-year survival rate for patients with metastatic disease is approximately 30 % [6].

The characterization of specific de-regulated genes in metastatic samples may help to define the tumor’s metastatic potential at a molecular level and to monitor disease progression as well as its response to therapy. Growing evidence indicates that normal DNA methylation patterns are altered in cancer cells as there is an overall decrease in the genomic content in 5-methylcytosine and frequent hypermethylation and inactivation of tumor suppressor genes [7]. Aberrant DNA methylation in candidate genes such as *FGFR1* [8], *JUP* [9], *MYOD1* [10], *PAX3* [11]*, RASSF1* [12]*, BMP2* [13] and *CAV1* [14] has also been described in RMS.

Microarray and novel sequencing techniques have facilitated the comprehensive analysis of the genome and have paved the way for genome-wide scanning of DNA methylation states [15]. Epigenetic information such as DNA methylation profiling could, in fact, help to identify tumor subtypes and lead to more accurate diagnoses [16–18]. Several genome-wide studies, which have demonstrated that distinct methylation patterns are found in ARMS vs ERMS and fusion-positive vs fusion-negative tumors [19–21], have shown that *PTEN* and *EMILIN1* are differentially expressed genes that may be regulated by DNA methylation.

The current study aimed to examine methylation patterns in alveolar and embryonal samples and to explore epigenetic changes in different RMS subtypes at various clinical stages. We delineated, for the first time, the association between metastatic phenotype and DNA methylation pattern. Study results also uncovered a novel gene whose expression is lowered by DNA methylation, suggesting that epigenetic therapy could be utilized to improve current treatment protocols of rhabdomyosarcoma.

## Methods

### Cell culture

Human ARMS (RH4 and RH30) and human ERMS cells (RD and RH36) were maintained in Dulbecco’s modified Eagle’s medium containing 10 % fetal calf serum, penicillin (100 U/mL), and streptomycin (100 ug/mL) (Life Technologies, Carlsbad, CA) at 37 °C in 5 % CO_2_ in a humidified incubator. RH30 and RD cells were obtained from American Type Culture Collection (Manassas, VA); RH4 were gift from Prof. Pier Luigi Lollini (Dept. Medicina Specialistica, Diagnostica e Sperimentale, University of Bologna, Italy) [22]. RH36 were obtained from Dr. Maria Tsokos (National Cancer Institute, Bethesda, MD) [23]. A summary of RMS cell line features is available in Additional file 1.

#### Tumor samples and ethics approval

Specimens were obtained from the Italian Association of Pediatric Hematology and Oncology Soft Tissue Sarcoma Bank at the Department of Women’s and Children’s Health, University of Padova (Padova, Italy). The study, part of a clinical trial carried out in association with the Association Italiana Ematologia Pediatrica AIEOP (Italian Association of Pediatric Hematology and Oncology), was approved by the local ethics committee. Selected clinical parameters of RMS patients used in the analysis are available in the Additional file 2.

### Total RNA and DNA isolation

Genomic DNA was isolated from RMS cell lines and from RMS tumor biopsies using Trizol® Reagent (Life Technologies) after RNA extraction following the manufacturer’s instructions. The commercially available Qiamp DNA mini Kit (Qiagen) was used to purify the DNA. Total DNA was quantified using the ND-1000 spectrophotometer (Nanodrop, Wilmington, DE).

#### Genome-wide DNA methylation profiles

Four μg of genomic DNA was fragmented by sonication and purified using Mini-Elute columns (Qiagen Co., Hilden Germany), and the amount of double-stranded DNA (dsDNA) was measured using the Qubit instrument (Invitrogen, Life Technologies Co., Carlsbad, CA, USA). The success of fragmentation was evaluated using the Agilent Bioanalyzer 2100 (Agilent Technologies, Santa Clara, CA, USA). The MethylMiner Methylated DNA enrichment kit (Invitrogen, Life Technologies Co., Carlsbad, CA, USA) was used to enrich the fraction of methylated dsDNA, starting from 2 μg of fragmented whole genomic DNA. Ten ng of methylated dsDNA for each sample was amplified using Whole Genome Amplification (WGA, Sigma-Aldrich Co., St. Louis, MO, USA). Genomic DNA was used as the control for each sample. DNA methylation profiling was carried out in RMS tumor samples using the *Human DNA Methylation Microarray* (Agilent Technologies, Santa Clara, CA, USA) consisting of about 244,000 (60-mer) probes designed to interrogate about 27,000 known CpG islands. The control genomic DNA and methylated dsDNA were labeled with Cy3 and Cy5 dye respectively using Agilent Genomic DNA labeling kit PLUS (Agilent Technologies, Santa Clara, CA, USA) and competitively hybridized to Human DNA Methylation microarrays platforms (GEO ID: GPL10878). The hybridization was carried out at 67 °C for 40 h in a hybridization oven rotator (Agilent Technologies, Santa Clara, CA, USA). The arrays were washed with Agilent ChiP-on-chip wash buffers as suggested by the supplier. Slides were scanned on an Agilent microarray scanner (model G2565CA), and Agilent Feature Extraction software version 10.7.3.1 was used for image analysis.

#### Availability of data and materials

Raw data are available on the GEO website using accession number GSE67201, and processed data are presented as Additional files 1, 2 and 3.

#### Statistical analysis of DNA methylation data

Intra-array normalization of methylation levels was performed with linear and lowess normalization. Inter-array normalization was performed with quantile normalization [24] in order to correct experimental distortions. The normalization function was applied to the methylation data of all the experiments. Feature Extraction Software (Agilent Technologies, Santa Clara, CA, USA) provided spot quality measures with regard to methylation expression data in order to evaluate the quality and the liability of the hybridization data. In particular, flag “glsFound” and “rlsFound” (set to 1 if the spot had an intensity value that was significantly different from the local background or to 0 in any other cases) were used to filter out unreliable probes: flag equal to 0 was to be noted as “not available (NA)”. Probes with a high proportion of NA values (more than 25 %) were removed from the dataset to ensure more robust, unbiased statistical analyses. When twenty-five percent of NA was used as the threshold in the filtering process, a total of 90.591 probes were obtained. The microarray data were analyzed using the iChip R bioconductor Package. The microarray data were processed in accordance with the instructions contained in the package vignette (www.bioconductor.org/packages/release/bioc/vignettes/iChip/inst/doc/iChip.pdf). Briefly, after normalization we computed the enrichment measure using the lmtstat function (a wrapper function of the empirical Bayes t-statistic from limma package) provided by iChip package. Specifically, we used the iChip2 function that implements the high order hidden Ising model described in [25]. The iChip2 function was called with *b* = 1 following the specifications for low resolution arrays, while the other parameters were left at the default value. iChip2 function Enriched regions were called using an FRD cutoff of 0.2 and maxGap = 500 bp.

The genes associated to DMRs identified using iChip algorithm were functionally analyzed using Gene Ontology (GO) implemented by the Database for Annotation, Visualization and Integrated Discovery (DAVID) tool [26]. The significantly enriched biological categories were identified using a Modified Fisher Exact *p*-value < 0.05.

### Trichostatin A and 5-aza-2′-deoxycytidine treatments

RMS cells (0.25x10^6^ cells/mL) grown in 100 mm dishes were treated with demethylating agent 5-aza-2′- deoxycytidine (5-Aza-dC) (Selleck Chemicals, Houston; TX, USA), with TSA (Selleck Chemicals, Houston; TX, USA), or with a combinatorial treatment using both agents. Concentrations varying from 100nM to 2 μM of 5-Aza-dC for 72 h and 200 ng/ml of TSA for 16 h were used. Cells were harvested and processed for RNA or DNA extraction.

### qRT-PCR for mRNA detection

For mRNA detection, 1 μg of total RNA was retrotranscribed with Superscript II (Life Technologies), and qRT-PCRs were carried out with gene-specific primers and the SYBR PCR Master Mix (Applied Biosystem, Life Technologies) using a ViiA 7 Real-Time PCR System.

*GADPH* was selected for the endogenous normalization of the gene expression analysis. The relative expression levels between samples were calculated using the comparative delta Ct (threshold cycle number) method (2^-ΔΔCt^) [27] implemented in the ViiA 7 Real-Time PCR System software. A 95 % confidence interval (IC) was calculated.

The relative expressions of non-clustered protocadherins (PCDHs) were simultaneously analyzed using the relative expression software tool (REST) which is able to identify significance differences between two groups of samples using a randomization test [28]. Permutation or randomisation tests are useful alternatives to more standard parametric tests because despite the fact that they remain as powerful as more standard tests, they make no distributional assumptions about the data. The randomisation test repeatedly and randomly reallocates the observed values to the two groups and notes the apparent effect (expression ratio in our case) each time. A proportion of these effects, which are as great as those actually observed in the experiment, gives the *P*-value of the test.

The statistical analysis of *PCDHA4* expression levels, evaluated in an expanded cohort of samples, was performed using Prism6 software, and the Mann–Whitney *U*-test was used.

#### Sodium bisulfite treatment of DNA and bisulfite sequencing

One μg of genomic DNA was subjected to conversion with sodium bisulfite using EZ DNA Methylation-Gold ™ kit (Zymo Research, Orange, CA, USA), following the manufacturer’s instructions. One hundred ng of bisulfite-converted DNA was used as template for the amplification of candidate regions. Polymerase chain reaction (PCR) was performed using methylation-independent primers designed with the free online tool MethPrimer (http:/itasa.ucsf.edu/~urolab/methprimer;). The PCR products were purified using the QIAquick PCR purification kit (Qiagen Co., Hilden Germany) and subcloned into pSC-A-amp/kan vector using the StrataClone PCR Cloning Kit (Agilent Technologies, Santa Clara, CA, USA). Competent cells were transformed with ligation reaction product and grown in Luria Bertani (LB) agar plates supplemented with 40 μg/ml of X-Gal (Promega Co., Madison, WI, USA) and 50 μg/ml of ampicillin for 16 h at 37 °C. Blue-white screening permitted identification of recombinant bacteria. Selected clones were evaluated by colony PCR performed using M13R and T7 universal primers (Invitrogen, Life Technologies Co., Carlsbad, CA, USA). The PCR products were checked for the presence of inserts using agarose electrophoresis, and those corresponding to positive clones were purified using a QIAquick PCR purification kit (Qiagen Co., Hilden Germany) and then sequenced by 3500 Dx Genetic Analyzer sequencer (Applied Biosystems, Life Technologies Co., Carlsbad, CA, USA) using BigDye® Terminator v3.1 CycleSequencing Kit (Applied Biosystems, Life Technologies Co., Carlsbad, CA, USA) following the manufacturer’s instructions.

#### RNA interference

RH36 cells at 50 % to 70 % confluence were transfected with small-interfering RNA (siRNA) for target gene *PCDHA4* (siPCDHA4) or with non-targeting siRNA (siCONTROL) using Lipofectamine2000 transfection reagent (Thermofisher Scientific). We performed preliminary experiments in the attempt to achieve the highest efficiency and reproducibility. The efficacy of gene knockdown was evaluated at the mRNA level using qRT-PCR analysis after 48 h of transfection.

#### Flow cytometric analysis of the cell cycle

After transfection, *PCDHA4* silenced cells (siPCDHA4) and control cells (siCONTROL) were harvested. For each sample, 1x10^6^ cells were fixed with 70 % cold ethanol, washed in PBS, and incubated with propidium iodide (50 μg/mL) and RNase (100 μg/mL) for 60 min at 37 °C. Samples were run in a BD FACScan (Becton Dickinson, Labware, Bedford, MA); the data were analyzed with ModFitLT V3.0 software (Verity Software House, Topsham, ME). Two independent experiments were performed with three replicates for each. A 95 % Confidence interval (CI) was calculated.

#### Invasion Transwell Assay

Chemoinvasion was measured using 24- well BioCoat Matrigel invasion chambers (Becton Dickinson) with an 8-μm pore polycarbonate filter coated with Matrigel. The lower compartment contained 0.5 mL of 1 % serum medium conditioned by the NIH3T3 cell line as a chemoattractant or serum-free Dulbecco’s modified Eagle’s medium as a control. In the upper compartment, 1x10^4^ RH36 cells per well were placed in triplicate wells and incubated for 18 h at 37 °C in a humidified incubator with a 5%CO2 atmosphere. After incubation, the cells on the filter’s upper surface were wiped off with a cotton swab; the cells on the lower surface were, instead, fixed in 2.5 % glutaraldehyde, stained with 0.2 % crystal violet in 20 % methanol, and then photographed using a stereomicroscope (model MZ16; Leica Microsystems) equipped with a charge-coupled device (CCD) camera. Images were processed using Corel-Draw software (Corel, Ottawa, Canada), and the area occupied by the migrated cells was measured using ImageJ software (http://rsbweb.nih.gov/ij, last accessed September 4, 2009). A 95 % Confidence interval (CI) was calculated.

## Results

### DNA methylation profiling in RMS tumor biopsies

We analyzed the DNA methylation profiles of 15 RMS samples - 6 *PAX3/FOXO1* positive ARMS, 3 *PAX3/FOXO1* negative ARMS and 6 ERMS - using the Human DNA methylation platform (Agilent) which is a collection of 244 k probes designed to interrogate about 27,000 known human CpG islands. We compared the methylation profiles of *PAX3/FOXO1* positive and negative RMS using the iChip R Bioconductor Package [25]. Analysis of the data (*false discovery rate* (FDR) <0.2) revealed that a set of differentially methylated regions (DMRs) were able to discriminate between the fusion positive and fusion negative RMS, as was previously demonstrated by Sun et al. [21] (Additional file 3).

We then used iChip R Bioconductor Package to compare the samples of disseminated and localized RMS and identified 1394 regions differentially methylated (FDR < 0.2) able to discriminate metastatic *vs* non-metastatic RMS (Additional file 3). We noted that the majority of DMRs (86.5 %) showed a positive enrichment value in the metastatic samples with respect that in the non-metastatic ones highlighting those genes’ tendency for hypermethylation in samples with metastatic disease. We then mapped the DMRs to the genome using the UCSC Genome browser and found that only 357 DMRs localize in promoter regions (defined as regions located from -2Kb + to 1 kb to transcription start site, TSS) while all the other DMRs mapped in intragenic regions or in CpG regions distal to known coding sequences (intergenic regions).

### Detection of novel methylated target genes in metastatic and non-metastatic samples

**Fig. 1:**
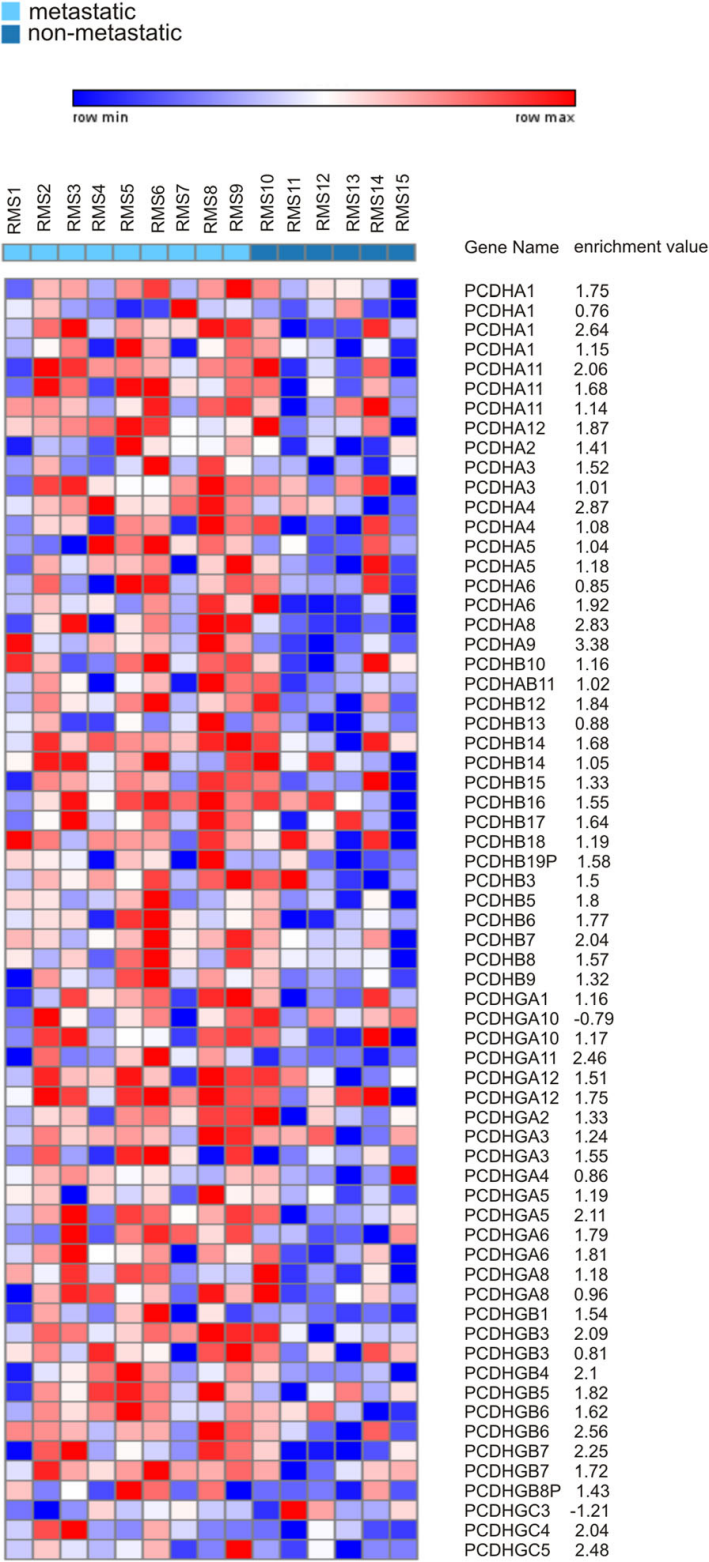
A heatmap of DMRs associated to *PCDHs* genes*.* The heatmap shows the DNA methylation patterns of protocadherin loci (measured as log2 ratio of methylated dsDNA/ total genomic DNA) for the RMS samples analyzed. Each column represents the profile of a specific RMS sample, and each row represents the differentially methylated regions (DMRs) associated to *PCDHs* genes identified with iChip algorithm by comparing metastatic and non-metastatic samples. The enrichment value, expressed as moderated t-statistics extracted using the eBayes function, is associated to each DMR as result of iChip analysis. A positive enrichment value indicates hypermethylation in metastatic vs non-metastatic samples

To detect genes directly or indirectly modulated by DNA methylation, we analyzed the genes associated to DMRs with the DAVID functional annotation tool which performs a GO-term analysis and identifies which functional categories are over-represented. The terms analyzed have a FDR-adjusted P value <0.05. We found a consistent number of genes involved in cell adhesion (45 genes, *P* = 6,7E10^−31^), cell-cell signaling (18 genes, *P* = 2.3E-02) and regulation of transcription (65 genes, *P* =4.8E10^−4^) which were the three major significantly enriched functional categories (Table 1). Interestingly, we found many members of the protocadherin clusters α,β,γ (PCDHs) in the cell adhesion category (Fig. 1). We also observed that all DMRs linked to protocadherins mapped in promoter regions. The finding was interesting given that several studies have demonstrated that some protocadherins play a tumor suppressor role in many cancer types [29].

**Table 1:** Summary and functional annotation of methylated genes over-represented in metastatic vs non-metastatic RMS samples

Biological Process ID	Terms	Count	*p*-value*	FDR
GO:0016337	cell-cell adhesion	45	6.70E-31	1.10E-27
GO:0007267	cell-cell-signalling	18	2.30E-02	3.2E + 01
GO:0006355	regulation of trancsription	65	4.80E-04	7.90E-01
GO:0048598	embryonic morphogenesis	16	1.90E-04	1.30E-02
GO:0022406	cell cycle phase	11	1.50E-01	9.30E + 01

The terms of enriched classes referred to biological processes identified by Gene Ontology (GO) classification performed using the DAVID web tool. FDR, false discovery rate. *Modified Fisher exact P-value identified by DAVID.

### Expression levels of protocadherins in RMS cell lines and tumor biopsies

**Fig. 2:**
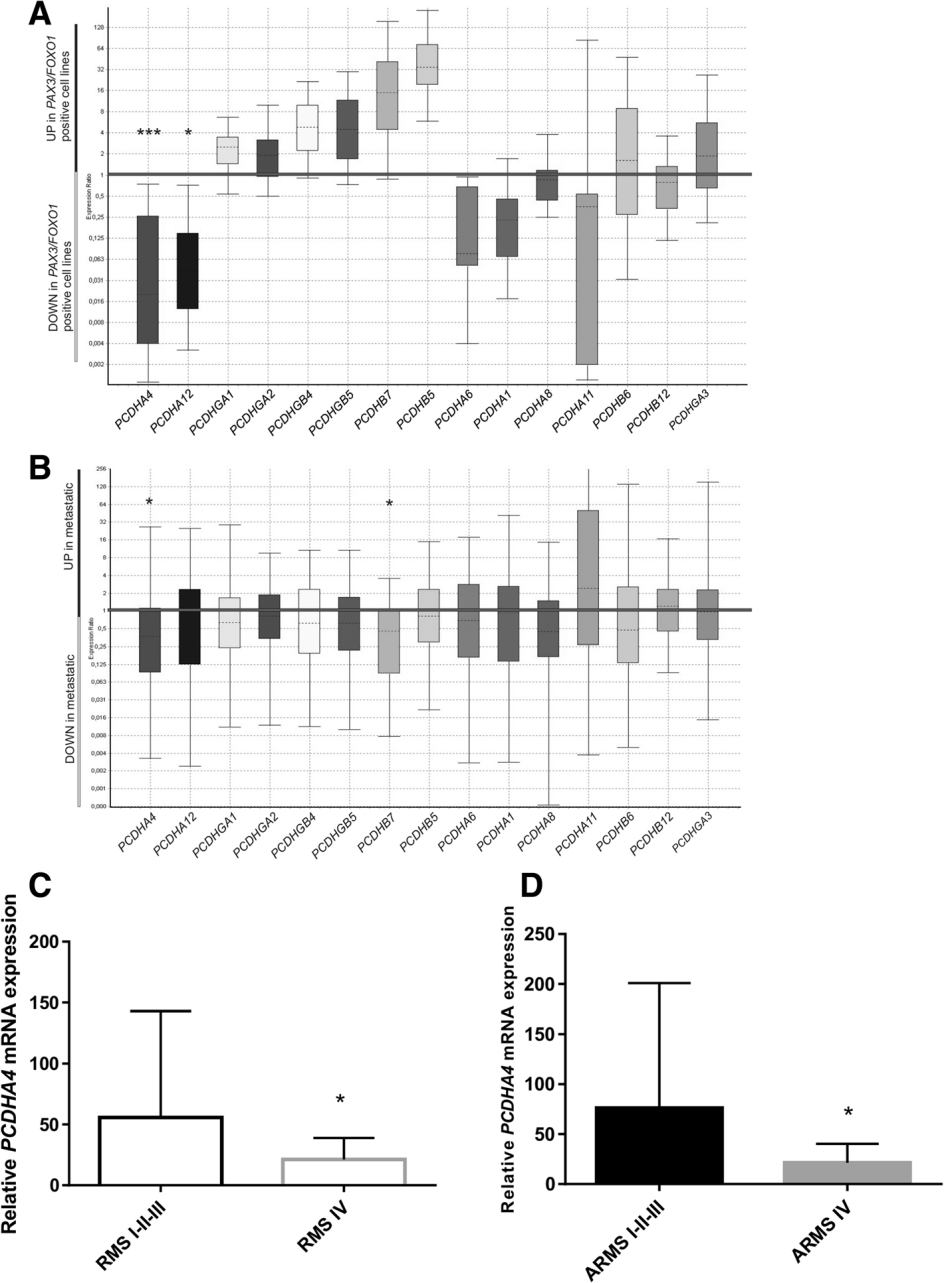
*PCDH*s genes expression level by qRT-PCR analysis. Relative expression levels of 15 *PCDH*s genes in *PAX3/FOXO1* ARMS cell lines compared to ERMS cell lines (**a**) and in metastatic RMS tumor samples compared to non-metastatic RMS tumor samples (*n* = 15) (**b**). Data distribution is represented by box plot analysis performed using REST software. The relative expression of the *PCDHA4* gene was evaluated in a larger cohort of RMS samples (*n* = 61). **c** *PCDHA4* mRNA levels were lower in metastatic RMS samples (stage IV) with respect to non-metastatic RMS (stage I-II-III). **d** Comparison of metastatic ARMS vs non-metastatic ARMS confirmed the association of low *PCDHA4* levels with the metastatic phenotype. Glyceraldehyde-3-phosphate dehydrogenase (GAPDH) was used as a housekeeping gene for data normalization. Relative expression (RQ) was calculated using ∆∆Ct method. Statistical analysis (Mann–Whitney *U*-test) was performed using Prism 6 software. * *P* < 0.05; **P < 0.01; ***P < 0.001

To study the involvement of protocadherins in RMS, we tested the gene expression of some members of PCDH α,β,γ clusters by qRT-PCR in both RMS cell lines and tumor biopsies. The expression data were analyzed using the relative expression software tool (REST) [28] which makes it possible to identify genes whose expression is different in two sample groups by applying a randomisation test. We analyzed 4 RMS cell lines, representative of the two major subtypes of RMS: alveolar *PAX3/FOXO1* positive RMS cells (RH4, RH30) and embryonal RMS cells (RD, RH36). We found significantly different expressions for *PCDHA12* (*P* = 0.021) and *PCDHA4* (*P* < 0.001) in the ARMS and ERMS cell lines (Fig. 2a), and we found statistically significant different expression levels of *PCDHA4* (*P* = 0.030) and *PCDHB7* (*P* = 0.004) in the metastatic tumor samples (IV stage) with respect to the non-metastatic ones (I-II-III stage) (Fig. 2b). Although several protocadherins resulted differentially expressed in the metastatic and non-metastatic RMS, only *PCDHA4* showed an opposite correlation between methylation status and gene expression. We then evaluated *PCDHA4* expression in a larger cohort of RMS samples (*n* = 61) and found that *PCDHA4* levels are higher in non-metastatic RMS (clinical stage I-II-III) with respect to metastatic at diagnosis RMS samples (IRS IV) (*p* < 0.05, Fig. 2c). A comparison of non-metastatic and metastatic ARMS provided further confirmation of the association between *PCDHA4* expression and clinical stage (*p* < 0.05, Fig. 2d).

### Restoration of PCDHA4 expression in RMS cells treated with 5Aza dC and trichostatin A

**Fig. 3:**
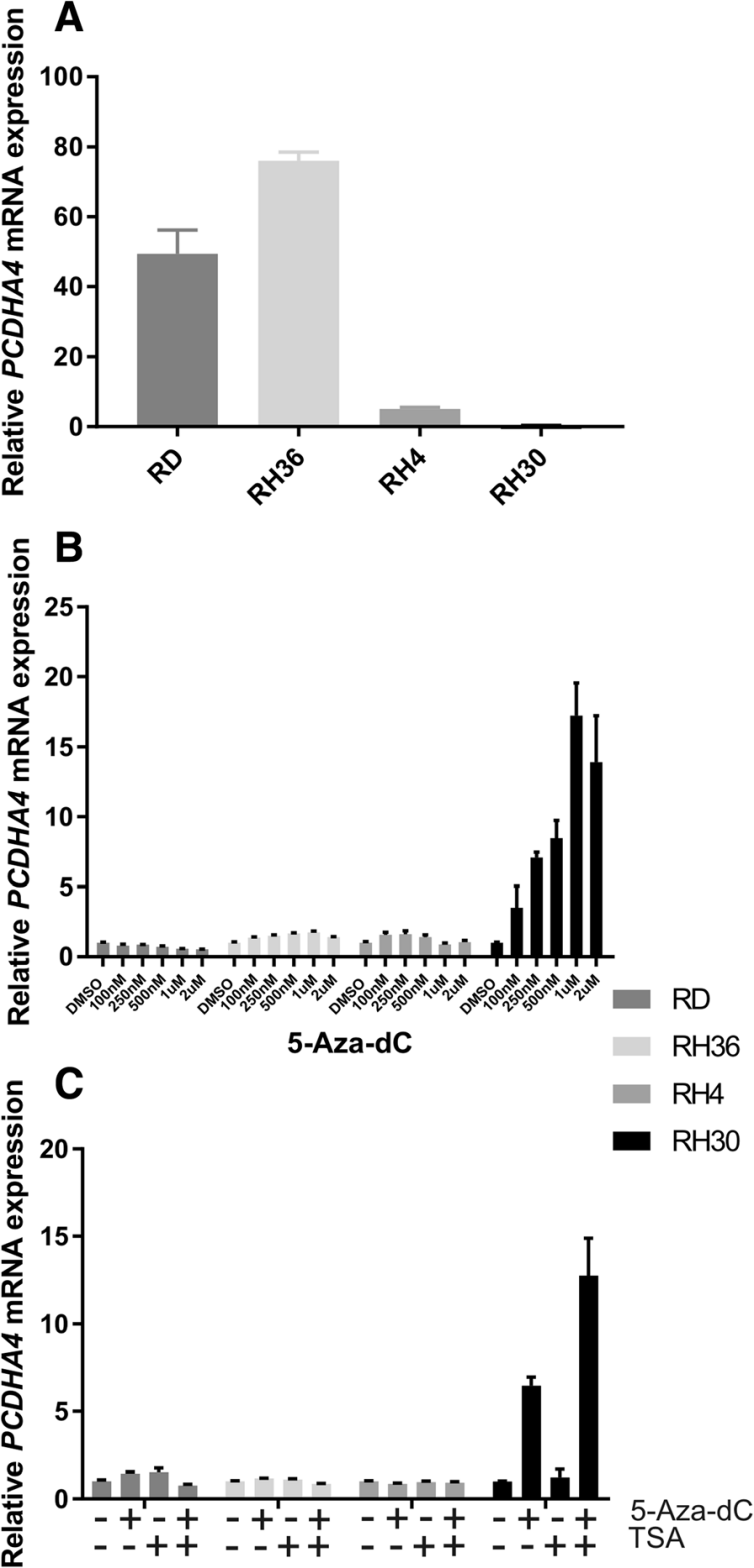
Relative expression of *PCDHA4* in RMS cells after treatment with 5-Aza-dC and/or TSA*.* The expression level of *PCDHA4* was evaluated in 4 RMS cell lines (**a**), in RMS cells after 72 h of treatment with increasing doses of 5-aza-dC (100 nM, 250 nM, 500 nM, 1 uM and 2 uM) (**b**) and in RMS cells after treatment with 1uM of 5-aza-dC (72 h), 200 ng/mL of trichostatin A (16 h) or a combination of both (**c**) by qRT-PCR. A housekeeping *GAPDH* gene was used as an internal control for normalization, and DMSO-treated cells were used as the calibrator. Relative expression was calculated using ∆∆Ct method. The error bar represents a 95 % confidence interval (IC). RH30, RH4: *PAX3/FOXO1* ARMS cell lines; RD, RH36: ERMS cell lines; 5-aza-dC: 5-aza-2′-deoxycytidine

The evaluation of *PCDHA4* expression levels in RMS cell lines by qRT-PCR revealed different expression patterns in ARMS (RH4 and RH30) and ERMS cell lines (RD and RH36) (Fig. 3a). In order to analyze whether DNA methylation can affect the gene expression in alveolar and embryonal RMS, we treated RMS cell lines with the demethylating agent 5-Aza-2′-deoxycytidine (5-Aza-dC) either separately or in conjunction with the histone deacetylase inhibitor Trichostatin A (TSA). It is known that histone acetylation/deacetylation is a central mechanism for regulating transcription through chromatin remodeling. Indeed, many studies have suggested that epigenetic cross-talk between DNA methylation and histone acetylation is involved in gene transcription and aberrant gene silencing in tumors. When we assessed *PCDHA4* expression level by qRT-PCR 72 h after treatment of increasing doses of 5-Aza-dC we did not observe any change in ERMS cell lines (RD and RH36), but we did notice a dose dependent restoration of *PCDHA4* expression in RH30, one of the ARMS cell lines representing the metastatic tumor (*p* < 0.05, Fig. 3b). Instead, while treatment with trichostatin A alone did not lead to of the restoration of *PCDHA4* expression, the combination of 5Aza-dC and TSA synergistically augmented mRNA expression of *PCDHA4* in ARMS cell lines RH30. Conversely, no effect on *PCDHA4* expression was observed in ERMS cell lines after combined treatments (Fig. 3c). Taken together, these results suggest that epigenetic regulation of *PCDHA4* may be present in RMS cells, and they underline the synergic effect of the two different drugs.

### Bisulfite sequencing confirms that PCDHA4 promoter has a different methylation pattern in RMS cell lines

**Fig. 4:**
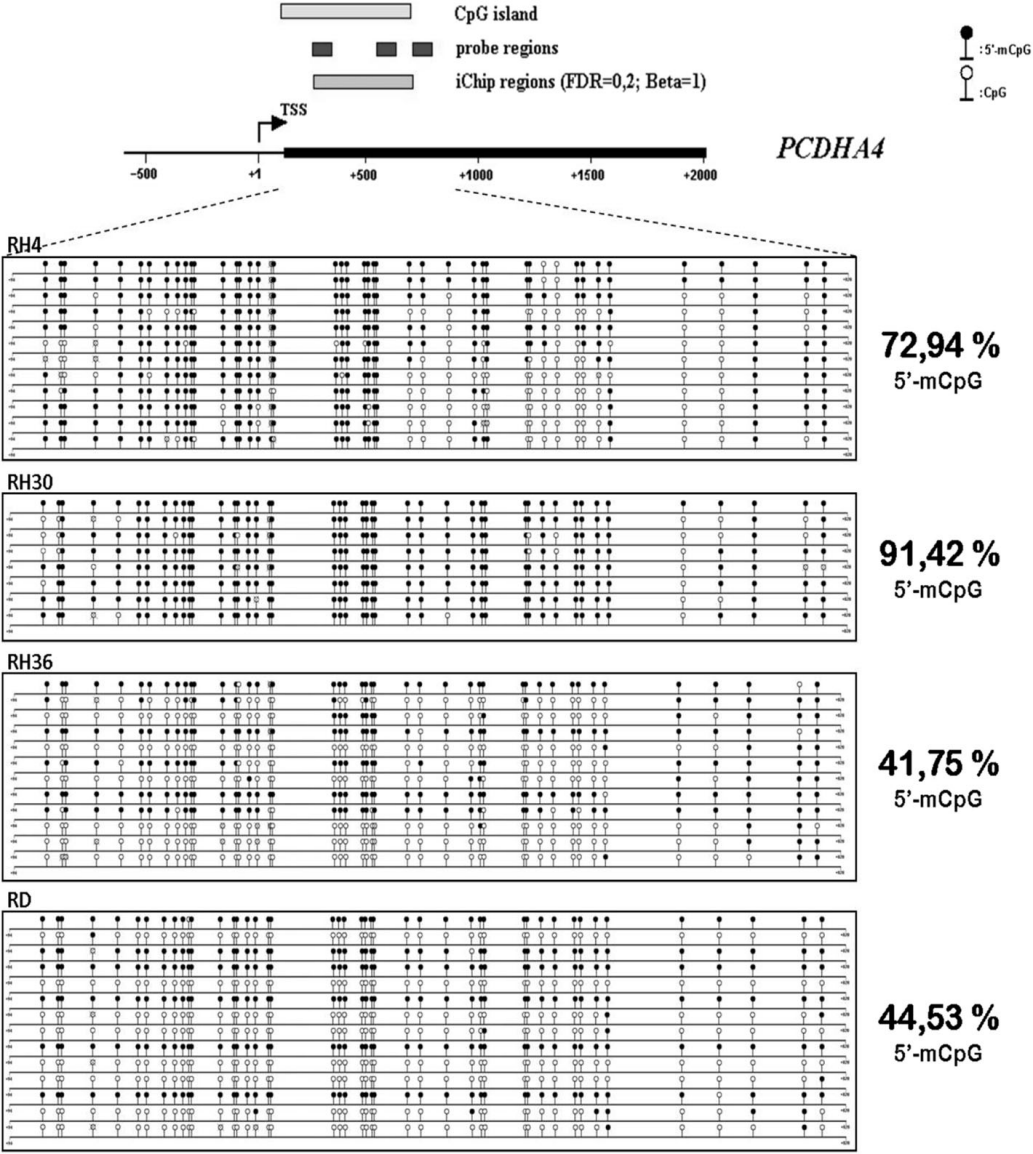
Sanger bisulfite sequencing of *PCDHA4* promoter region revealed different methylation patterns in *PAX3/FOXO1* ARMS cell lines and ERMS cells. Sequencing was performed for at least 8 clones obtained by subcloning bisulfite-converted promoter region. Sequenced region spanned from position +94 to +828, where position +1 corresponds to the gene transcription start site (TSS). The sequence investigated maps on a predicted CpG island and includes the region identified with iChip algorithm. ARMS cell lines have high methylation levels (RH4, RH30) with respect to ERMS cell lines (RH36, RD). Circles: cytosine within CpG dinucleotides; black circles: methylated cytosine; white circles: unmethylated cytosine

To verify the promoter methylation status of *PCDHA4,* we performed bisulfite Sanger sequencing in four RMS cell lines. As above, we used two positive ARMS cell lines (RH4 and RH30) and two ERMS cell lines (RD and RH36). We designed a bisulfite sequencing assay inside the CpG island that overlaps the promoter of *PCDHA4* and also contains the DMR identified by microarray experiments (Fig. 4). The bisulfite-converted DNA region was amplified by PCR and subcloned into bacterial vector. We then performed Sanger sequencing of at least eight clones obtained by subcloning the amplified *PCDHA4* putative promoter region. Bisulfite sequencing data showed that the methylation level was higher in the ARMS (72.94 % of 5′m-CpG in RH4 and 91.42 % of 5′m-CpG in RH30) than in ERMS cell lines (41.75 % of 5′m-CpG in RH36 and 44.53 % of 5′m-CpG in RD) (Fig. 4). We thus confirmed that different methylation levels of *PCDHA4* promoter regions characterize RMS cell lines originating from metastatic and non-metastatic tumors.

### PCDHA4 involvement in tumor growth of RMS cells

**Fig. 5:**
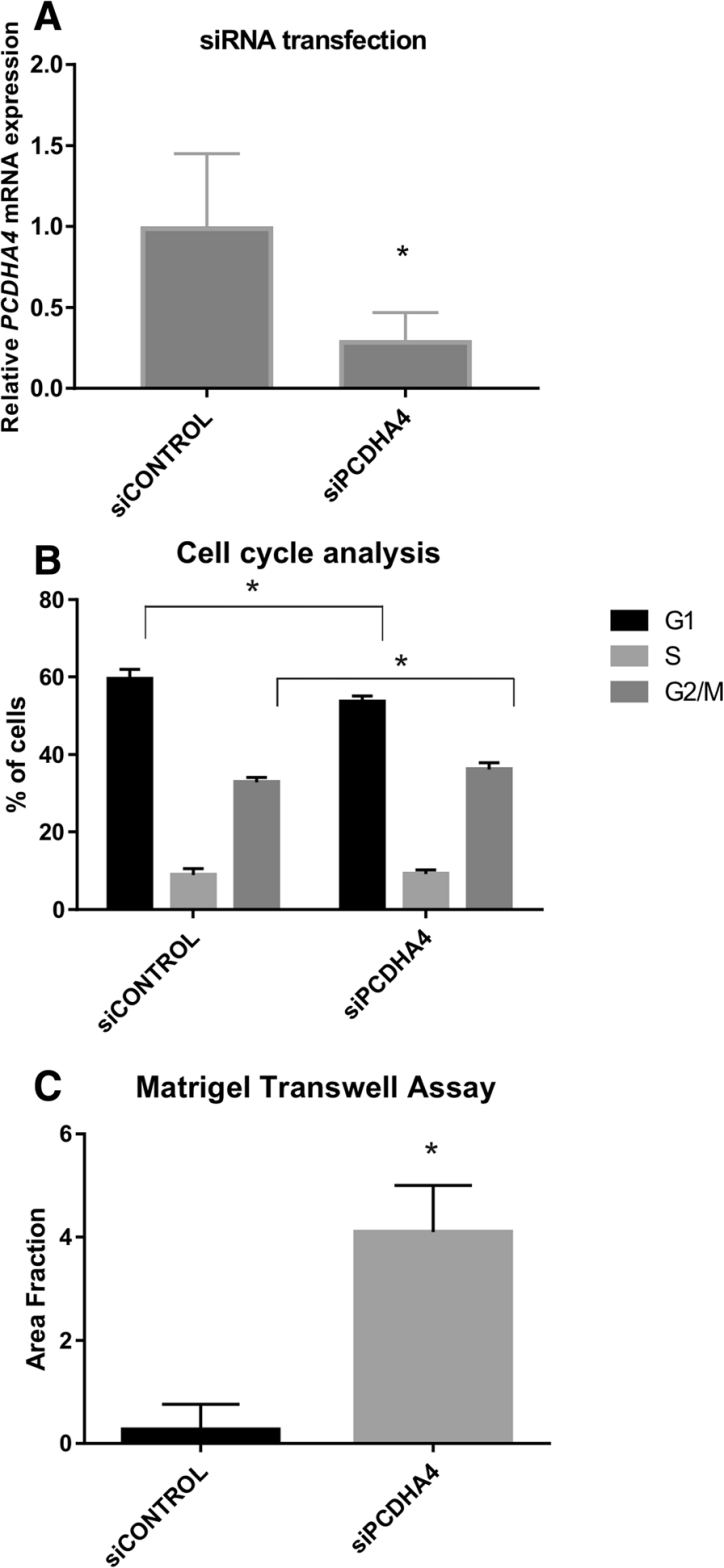
*PCDHA4* involvement in tumor cell growth and invasion*.* RH36 cells that express high levels of *PCDHA4* were transiently transfected with siRNA for *PCDHA4* (siPCDHA4) or negative siRNA control (siCONTROL). The efficiency of silencing was evaluated using qRT-PCR (**a**). Transfected cells were used to analyze cell cycle distribution (**b**) or invasion **c**). The results are shown as mean ± IC of the percentage (%) of proliferation, measured using flow cytometry 48 h after transfection (**b**) and as the area covered by Matrigel-invading cells (**c**). Cell proliferation and invasion of *PCDHA4* silenced cells are shown relative to control values (siCONTROL). Two independent experiments were performed in triplicate and mean results are shown. *P < 0.05; (The error bar represent a 95 % confidence interval, IC)

To evaluate the potential role of *PCDHA4* as a tumor suppressor in RMS, we transiently silenced *PCDHA4* in RH36, which is the ERMS cell line with the highest endogenous expression level of this gene. Transient modulation was evaluated 48 h post-transfection for expression and for biological effects. We observed an approximate 70 % reduction in gene expression when we used a qRT-PCR assay (Fig. 5a). Cell cycle progression of the transfected cells was assayed by flow cytometry. An increase in the proliferation rate as well as a G0/G1 phase decrease were observed in the cells with low levels of *PCDHA4* (siPCDHA4) with respect to what was noted in the control cells (siCONTROL) (Fig. 5b). Moreover, when invasion through Matrigel was evaluated by transwell assays, we noted enhanced mobility of *PCDHA4* silenced cells (siPCDHA4) with respect to that in controls (siCONTROL) (Fig. 5c). Taken together these preliminary results suggest that *PCDHA4* could play the role of a tumor suppressor and may be involved in promoting cell cycle progression and cell invasion of RMS cells.

## Discussion

During the current study, methylation profiling of RMS samples using a genome-wide approach uncovered differences in DNA methylation signatures of metastatic and localized RMS and highlighted that epigenetic alterations are peculiar to disseminated RMS. The findings demonstrated that DNA methylation can contribute to defining the molecular features of RMS subgroups and can thus increase the accuracy of RMS subtype classification. *PCDHA4* was identified as a gene whose expression was decreased in metastatic with respect to non-metastatic RMS. Preliminary data also suggested that *PCDHA4* may act as a tumor suppressor in RMS cells and that it may be partially inactivated by DNA methylation.

Several investigators including ourselves have examined the different behaviors and molecular features of alveolar and embryonal RMS, and over the past decade many studies have confirmed that gene expression profiles distinguish between alveolar *PAX3/FOXO1* positive ARMS and *PAX3/FOXO1* negative ARMS and ERMS [3, 4]. Although the mechanisms underlying different gene regulation patterns are still under investigation, it is known that epigenetic modifications, and in particular DNA methylation, can modulate gene expression and represents a challenging area of cancer research. Recent studies have demonstrated that DNA methylation profiles distinguish between RMS fusion-positive and negative samples and could be used to improve the molecular classification of rhabdomyosarcoma [19, 21].

Analysis of our microarray data confirm that alveolar *PAX3/FOXO1* positive samples have a different methylation signature with respect to RMS fusion-negative ones. It is important to remember that our analysis of DNA methylation differences in metastatic and non-metastatic tumors is based on the Intergroup Rhabdomyosarcoma Study (IRS) grouping which is highly predictive of tumor outcome. In particular, patients classified as IRS IV group, which is characterized by metastatic disease, have long-term failure-free survival (FFS) rates of <30 % [6, 30]. When we compared non-metastatic (IRS I-II-II) with metastatic (IRS IV) RMS patients, we found quite distinct methylation signatures. This data, together with those produced by other genome-wide studies highlight methylation pattern changes in different RMS subtypes and seem to suggest that an epigenetic therapy could be appropriate to treat rhabdomyosarcoma. Indeed, DNA methylation is an excellent target for anti-cancer therapy as it involves a reversible process that does not affect the DNA sequence.

No epigenetic drugs are currently included in RMS clinical protocols, but over the past few years, several new anti-cancer drugs with epigenetic activities have received approval for clinical trials on other solid cancers leading to a detailed characterization of their mechanisms of action. Some FDA-approved drugs for the treatment of solid cancers are 5-Aza that target histone deacetylase (HDAC) in metastatic non-small cell lung cancer [31] and inhibitors of EZH2, a subunit of Polycomb repressive complex in diffuse large B cell Lymphoma (DLBCL) [32].

When we analyzed the distribution in the genome of the differentially methylated regions (DMRs) identified by microarray analysis, we found that only 25 % of them overlap with promoter regions (regions defined as 2 kb upstream and 1 kb downstream of RefSeq transcription star site) while the others map inside gene bodies or are localized in intergenic regions distal to known genes. Advances in genome-wide approaches have demonstrated that methylation varies depending upon the specific genomic context. Although the majority of studies have focused on methylation in the promoter region adjacent to the transcription start site (TSS), methylation of the gene body or intergenic regions seems to have a functional role and contributes to defining the whole picture of the methylation status [33].

Our microarray analysis revealed an abnormal methylation pattern in promoters of protocadherins (PCDHs). PCDHs, which are a group of transmembrane proteins, constitute the largest subfamily of the cadherin cell-adhesion molecules. In mammals, PCDHs are organized in clusters (α,β,γ) or are scattered throughout the genome [29, 34]. The methylation value [the enrichment value expressed as moderated t-statistics computed using the eBayes function (limma-t)] of DMRs associated to protocadherins was that of a common hypermethylation level of promoter regions in metastatic compared to non-metastatic samples. Using qRT-PCR assays, we analyzed the expression levels of some PCDHs and observed that the correlation between promoter hypermethylation and downregulation of genes is very low, indicating that epigenetic alterations may have an alternative effect with respect to typical gene regulation. Other genome wide studies have reported the same result suggesting that the correlation between DNA methylation and mRNA expression does not always conform to the paradigmatic inverse correlation between the two processes [21].

Our findings highlighted *PCDHA4* as an example of a gene in RMS whose expression differs in metastatic and non-metastatic RMS samples. We hypothesize that the decrease in *PCDHA4* expression depended on DNA methylation. Interestingly, a recent methylation profiling study of cervical cancer samples revealed a methylation silencing of many clustered protocadherins in the cancer with respect to the control cells. The study also reported that there was a positive correlation between methylation frequency of *PCDHA4* and *PCDHA13* and tumor severity, highlighting the role of *PCDHA4* silencing in cancer progression [35]. Other studies have demonstrated the involvement of several protocadherins in tumor processes. It has been shown that protocadherins behave as tumor suppressor genes in many solid cancers such as non-small-cell lung cancer, gastric and prostate cancer. It has also been demonstrated that their involvement is due to aberrant DNA methylation that determines an altered expression pattern [36–38].

In the light of these findings, we performed some in vitro functional studies on RMS cells. These preliminary data uncovered the potential role of *PCDHA4* as a tumor suppressor in RMS. In fact, we observed that *PCDHA4*-silenced cells acquire a more aggressive phenotype, as demonstrated by the increase in proliferation rate and invasiveness that was found. Further functional studies are warranted to clarify the involvement of *PCDHA4* in rhabdomyosarcoma.

Treatment with 5-Aza-dC and/or TSA of RMS cell lines suggested that there is epigenetic control of *PCDHA4* expression. Although the restoration of *PCDHA4* expression was noted only in one alveolar cell line (RH30), no changes in *PCDHA4* expression levels were observed in embryonal cell lines (RD, RH36). Bisulfite sequencing thus confirmed the different methylation status of the *PCDHA4* promoter region in alveolar and embryonal cell lines. Taken together, these data suggest that DNA methylation probably decreases the transcription of *PCDHA4* selectively in a RMS subgroup. Our experiments also demonstrated that a combination of 5-aza-2′-deoxycytine and trichostatin A drugs in RMS cells act synergistically to restore *PCDHA4* expression confirming the potential utility of a combination therapy. The combination of demethylating molecules with drugs that target histone modifications may enhance the efficacy of treatment as already demonstrated by other studies [39, 40].

## Conclusion

The current study has demonstrated for the first time that DNA methylation patterns differ in metastatic and non-metastatic RMS, and it has confirmed that epigenetic changes characterize rhabdomyosarcoma subtypes. In vitro treatment of RMS cells with demethylating agent 5-Aza-dC alone or together with histone deacetylase inhibitor suggests that there is epigenetic control of the gene regulation of *PCDHA4*. These findings were supported by bisulfite sequencing of *PCDHA4* promoter in RMS cells. Preliminary functional studies also suggested that *PCDHA4* could play a tumor suppressor role since it was hypermethylated and silenced in alveolar RMS cell lines representative of a metastatic tumor. Taken together, the findings point to a potential biomarker and a possible new therapeutic target for metastatic RMS.

## Additional files

Additional file 1: Summary of features of RMS cell lines (PDF 4 kb)
Additional file 2: Summary of the clinical characteristics of the RMS patients analyzed (PDF 36 kb)
Additional file 3: Results of DNA microarray data analysis using iChip. The main output of iChip is a bed file format that we modified to include the reference gene for the enriched region and the reference probe enrichment score (the probe with the largest enrichment value in the region). More specifically, the fields are the following: chromosome (chr), genomic start and end position of the enriched region (gstart and gend respectively), the gene name associated to the probe with the largest enrichment value (gene), the enrichment value expressed as moderated t-statistics using eBayes function (limma-t), the row number of *gstart* and *gend* in the position matrix (rstart and rend respectively), the genomic position where the probe has the largest enrichment value (peakpos), the mean posterior probability of the probes in the enriched region (meanpp), the maximum posterior probability of the probes in the enriched region (maxpp) and the number of probes in the enriched region (nprobe). (XLS 242 kb)

## Abbreviations

5-aza-dC: 5-aza-2′-deoxycytine
ARMS: Alveolar rhabdomyosarcoma
CpG: Cytosine-phosphate-guanine nucleotide sequence
DMRs: Differentially methylated regions
DNMT: DNA methyltransferase inhibitors
dsDNA: Double strand DNA
ERMS: Embryonal rhabdomyosarcoma
GO: Gene ontology
HDAC: Histone deacetylase inhibitors
PCDHs: Protochaderins
qRT-PCR: Quantitative real time polymerase chain reaction
RMS: Rhabdomyosarcoma
TSA: Trichostatin A
