# Supplementary material for: Global DNA methylation profiling uncovers distinct methylation patterns of protocadherin alpha4 in metastatic and non-metastatic rhabdomyosarcoma
Source: BMC Cancer. 2016 Nov 14;16:886. doi: 10.1186/s12885-016-2936-3 (PMC5109816; doi:10.1186/s12885-016-2936-3)
Supplement: Additional file 1: — Summary of features of RMS cell lines (PDF 4 kb) [file 12885_2016_2936_MOESM1_ESM.pdf]

Cell lines	Karyotype/Gene fusion status	Hystology	Origins
**RH4**	t(2;13)(p25;q14); TP53 mutation	Alveolar	Lung metastasis 7-year-old female
**RH30**	t(2;13)(p25;q14); TP53 mutation; amplification of 12q13-15 region including CDK4	Alveolar	Bone marrow metastasis 16-year-old male
**RH36**	Unknow	Embryonal	Paratesticular relapse 15-year-old male
**RD**	51-hyperdiploid; MYC amplification; Q61H mutation of NRAS; TP53 mutation	Embryonal	Pelvic mass 7-years-old female
