# Supplementary material for: Global DNA methylation profiling uncovers distinct methylation patterns of protocadherin alpha4 in metastatic and non-metastatic rhabdomyosarcoma
Source: BMC Cancer. 2016 Nov 14;16:886. doi: 10.1186/s12885-016-2936-3 (PMC5109816; doi:10.1186/s12885-016-2936-3)
Supplement: Additional file 2: — Summary of the clinical characteristics of the RMS patients analyzed (PDF 36 kb) [file 12885_2016_2936_MOESM2_ESM.pdf]

RMS	Translocation	Gender	Histology	IRS
RMS1	PAX3/FOXO1	Female	Alveolar RMS	Group IV
RMS2	PAX3/FOXO1	Female	Alveolar RMS	Group IV
RMS3	PAX3/FOXO1	Male	Alveolar RMS	Group IV
RMS4	NO	Female	Embryonal RMS	Group IV
RMS5	PAX3/FOXO1	Male	Alveolar RMS	Group IV
RMS6	PAX3/FOXO1	Female	Alveolar RMS	Group IV
RMS7	NO	Male	Alveolar RMS	Group IV
RMS8	NO	Male	Embryonal RMS	Group IV
RMS9	NO	Male	Embryonal RMS	Group IV
RMS10	NO	Female	Alveolar RMS	Group II a
RMS11	NO	Female	Alveolar RMS	Group III a
RMS12	NO	Male	Embryonal RMS	Group III b
RMS13	NO	Male	Embryonal RMS	Group III b
RMS14	NO	Female	Embryonal RMS	Group III a
RMS15	PAX3/FOXO1	Male	Alveolar RMS	Group III a
RMS16	PAX3/FOXO1	Female	Alveolar RMS	Group IV
RMS17	NO	Female	Embryonal RMS	Group III a
RMS18	PAX3/FOXO1	Female	Alveolar RMS	Group IV
RMS19	PAX3/FOXO1	Female	Alveolar RMS	Group IV
RMS20	PAX3/FOXO1	Female	Alveolar RMS	Group III a
RMS21	NO	Male	Embryonal RMS	Group II a
RMS22	PAX7/FOXO1	Female	Alveolar RMS	Group IV
RMS23	NO	Male	Embryonal RMS	Group III a
RMS24	NO	Female	Embryonal RMS	Group III a
RMS25	PAX3/FOXO1	Female	Alveolar RMS	Group III a
RMS26	PAX3/FOXO1	Male	Alveolar RMS	Group III a
RMS27	PAX3/FOXO1	Female	Alveolar RMS	Group IV
RMS28	NO	Male	Embryonal RMS	Group IV
RMS29	NO	Male	Embryonal RMS	Group III a
RMS30	NO	Female	Embryonal RMS	Group III a
RMS31	NO	Male	Alveolar RMS	Group III b
RMS32	PAX7/FOXO1	Male	Alveolar RMS	Group III a
RMS33	PAX7/FOXO1	Male	Alveolar RMS	Group III a
RMS34	NO	Male	Alveolar RMS	Group III a
RMS35	NO	Male	Alveolar RMS	Group III b
RMS36	NO	Female	Embryonal RMS	Group III a
RMS37	NO	Female	Embryonal RMS	Group III a
RMS38	NO	Female	Embryonal RMS	Group III a
RMS39	NO	Male	Embryonal RMS	Group III a
RMS40	PAX7/FOXO1	Female	Alveolar RMS	Group III a
RMS41	PAX3/FOXO1	Female	Alveolar RMS	Group IV
RMS42	NO	Female	Alveolar RMS	Group III a
RMS43	PAX7/FOXO1	Female	Alveolar RMS	Group III a
RMS44	NO	Male	Embryonal RMS	Group III a
RMS45	PAX3/FOXO1	Male	Alveolar RMS	Group IV
RMS46	PAX3/FOXO1	Female	Alveolar RMS	Group IV
RMS47	NO	Male	Embryonal RMS	Group III
RMS48	NO	Female	Embryonal RMS	Group III a
RMS49	NO	Male	Embryonal RMS	Group III b
RMS50	NO	Male	Embryonal RMS	Group III a
RMS51	PAX3/FOXO1	Female	Alveolar RMS	Group III a
RMS52	NO	Male	Embryonal RMS	Group III a
RMS53	PAX3/FOXO1	Male	Alveolar RMS	Group III a
RMS54	PAX3/FOXO1	Male	Alveolar RMS	Group IV
RMS55	PAX3/FOXO1	Female	Alveolar RMS	Group IV

RMS56	NO	Female	Alveolar RMS	Group III a
RMS57	NO	Female	Embryonal RMS	Group IV
RMS58	PAX3/FOXO1	Female	Alveolar RMS	Group IV
RMS59	PAX7/FOXO1	Male	Alveolar RMS	Group III a
RMS60	NO	Male	Embryonal RMS	Group III a
RMS61	NO	Female	Alveolar RMS	Group III a

* RMS cases analyzed by array

IRS Clinical grouping classification
